# Resolution of Otitis Media with Effusion in Adults after a Three-Day Course of Treatment with a Manosonic Nebulizer—A Pilot Study

**DOI:** 10.3390/medicina59020201

**Published:** 2023-01-19

**Authors:** Katarzyna Zasadzińska-Stempniak, Bartosz Karwat, Natalia Jarmołowicz-Aniołkowska, Hanna Zajączkiewicz

**Affiliations:** Department of Otorhinolaryngology, Head and Neck Diseases, School of Medicine, University of Warmia and Mazury in Olsztyn, al. Warszawska 30, 10-082 Olsztyn, Poland

**Keywords:** otitis media with effusion, Eustachian tube disfunction, insufflation

## Abstract

*Background and Objectives*: Aerosol drug administration is the primary treatment modality of otitis media with effusion (OME). An automatic manosonic aerosol generator (AMSA) delivers, with an acoustic overpressure, a therapeutic dosage of a drug by inhalation of the aerosol. However, available studies confirming their efficacy, especially in adults, are limited. Therefore, this pilot single-arm trial aimed to analyze changes in adults with OME following AMSA treatment. *Materials and Methods*: A group of 36 patients (mean age 51.4 years) with OME underwent a three-day treatment with inhaled mucolytic and steroids administered by AMSA. Tympanometry (tympanogram type, volume, compliance, pressure, and gradient) was performed to measure middle ear effusion before and after the intervention. *Results*: Following the intervention, partial and complete OME remission was observed in, respectively, 29 (81%) and 14 (39%) patients. The tympanogram type of the affected ears differed between baseline and after intervention measurements (*p* < 0.001). Tympanometry-based normalization, improvement deterioration and no change were observed in, respectively, 34 (68%), 1 (2%) 2 (4%), and 13 (26%) affected ears. Following the intervention, we observed an increase in continuously assessed middle ear volume (∆_median_ 0.19 mL, *p* = 0.002) and pressure (∆_median_ 142 daPa, *p* < 0.001), as well as a higher proportion of patients achieving categorical normalization of compliance (16% vs. 54%, *p* < 0.001) and pressure (28 vs. 64%, *p* < 0.001). *Conclusions*: Treatment efficacy was not affected by age, sex, or season of recruitment (all *p* > 0.05). The results of this pilot study are encouraging, however, the use of AMSA management of OME in adults needs to be verified in future studies.

## 1. Introduction

Aerosol drug administration is a possible treatment modality of otitis media with effusion (OME). Inhalation therapy aims to deliver a therapeutic dosage of a drug by inhalation of the aerosol, which is generated from a drug solution or suspension by a spray applicator or nebulizer [1]. In nebulizers, the sonic function produces a sound wave to the aerosol, which enhances penetration and drug deposition. in addition, an acoustic overpressure is generated in the nasal cavity by the device’s additional manosonic function. Such a system with that additional function is called an automatic manosonic aerosol (AMSA) generator [1]. The first AMSA device was constructed in France almost 30 years ago [2].

The primary concept of AMSA functioning was based on rehabilitating the interacting muscles during swallowing. First, velar muscles assist in nasopharyngeal tightness, then subsequent contraction of the uvular muscles opens the Eustachian tube (ET). The manosonic effect is automatically produced in the nose at the exact moment of swallowing. During the ET opening, acoustic vibration delivers air and nebulized drug into the tympanic cavity. The middle ear pressure is equalized, and the surrounding muscles’ strength and tonus are developed. Consequently, the ET adapts and preserves permanent effects by decreasing the OME symptoms [3].

The initial indications for AMSA use included ET dysfunction-related diseases, such as OME in adults and children over three years of age [3]. Other possible applications are the retraction pocket, adhesive otitis, tube failure following tympanoplasty, as well as rhinosinus pathology, especially in maxillary sinuses [2,3].

Despite a wide range of potential applications, current guidelines do not cover the use of AMSA. The only direct mention of AMSA was made in the “Consensus document for prescription of nebulization in rhinology”, where it is a strong agreement that nebulizers with additional sonic vibration are recommended in rhinosinus pathology [1]. Other guidelines refer to AMSA-like devices indirectly, as they can be categorized as insufflation methods with a modified Politzer test based on their mechanism of action.

In 2015, the American Academy of Otolaryngology published guidance for the management of OME. The group strongly does not recommend intranasal and systemic steroids, antibiotics, antihistamines, and decongestants, as they have been proven ineffective in OME treatment [4]. The body of evidence for the efficacy of insufflation methods is insufficient. According to NICE (National Institute for Health and Care Excellence), insufflation (e.g., Otovent device) may be considered a first-line option during or after the watchful waiting period [5]. An open randomized study demonstrated moderate improvement among children with OME aged 4–11 years after insufflation therapy [6]. On the other hand, recently published results on the insufflation method were ambiguous [7]. Although a few clinical trials provided evidence for the efficiency of AMSA in OME, they included small and highly selective groups, which excluded adults [8,9,10]. Moreover, these studies were not randomized and had no long-term follow-up [8,9,10]. By contrast, the drug protocols for manosonic nebulizers are not unified, and the duration of therapy is unclear. There seems to be an unfilled gap in the literature concerning efficacy and the protocol of AMSA use in adults.

Therefore, this pilot study aimed to analyze tympanometry changes in adults with OME following a three-day course of mucolytic and steroid administration with AMSA.

## 2. Materials and Methods

### 2.1. Study Design

We conducted a pilot, single-arm trial. Our study population comprised 36 patients recruited from the Outpatient Clinic of the Department of Otorhinolaryngology, Head and Neck Diseases in Olsztyn, Poland. All patients provided written informed consent. Patients were included if they filled the following criteria:

(1) Otologic diagnosis of unilateral or bilateral OME in the otoscopy at the visit before the recruitment;
(2) At least unilateral type B, C1, or C2 tympanogram;
(3) Aged between 18 and 70 years.

The exclusion criteria were:

(1) Otologic diagnosis of acute otitis media and other ear abnormalities at the visit before the recruitment;
(2) History of the active neoplastic disease;
(3) Current pregnancy;
(4) History of adenoid hypertrophy or nasal polyps.

Ethical approval was obtained from the ethics committee of the University of Warmia and Mazury (reference number 16/2017). All participants gave written informed consent.

**Figure 1:**
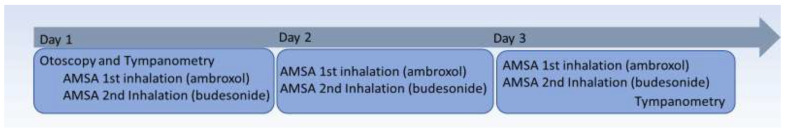
A diagram illustrating the setting of the AMSA protocol in this study.

Baseline characteristics of patients (age, gender, date of initial outpatient clinic visit), as well as prior medical history, were extracted from the electronic patient dataset. We collected data on adverse effects occurring during the administration of the treatment using a standard questionnaire. Nasal endoscopy was made by board-certified otolaryngologists with at least 15 years of experience. Tympanometry and otoscopy findings were recorded before and after the intervention. A normal tympanic membrane was diagnosed when no gas bubbles, exudate, or retraction were present. A pathological eardrum was identified when bubbles, middle ear exudate, or tympanic membrane retraction were noted. The intervention structure and procedures are shown in Figure 1.

### 2.2. Intervention

The study intervention consisted of 3 days of inhalation (2 inhalations at each visit) with an AMSA device (ATOMISOR^®^—PURENEB^®^, DTF Medical, Saint-Etienne, France).

An atomizer, with a capacity of 12 mL, is a hand-held device fitted with a binary nasal adapter. It produces from an aerosol fluid with a particle size of 2.2 µm, emitted at a flow rate of 0.20 mL per minute [10]. The atomizer is attached to the device by two tubes, one transmitting sonorous vibrations (100 Hz sound waves) and the other sending pressure pulses. A nasal piece (PURENEB^®^ MS1A/E nebulizing kit) is inserted into the nostrils. At the first visit, before the intervention, participants were instructed by a team member on how to use the device. A patient has to swallow upon request, initiating the process and releasing overpressure to the nasal cavity. The value of the overpressure can be adjusted through a graduated knob from 15 mbar to 50 mbar and is regulated individually by the patient.

For the first inhalation, the aerosol was obtained from a solution composed of: 2.5 mL of ambroxol solution for nebulization (7.5 mg/mL) mixed with 1 mL of 0.9% saline. The second inhalation’s aerosol consisted of a suspension of 2 mL budesonide (0.25 mg/mL) mixed with 1 mL of 0.9% saline. In total, patients received 1.5 mg of budesonide and 56.25 mg of ambroxol by nebulization during three days of treatment.

### 2.3. Tympanometry

Tympanometry was performed before and on after the intervention (the third day of the inhalation). Tympanograms were obtained using the Interacoustics Titan Clinical device and Titan Suite Software (Interacoustics A/S, Middelfart, Denmark). We measured the following parameters: volume (normal range: <2.5 mL), compliance (normal range: 0.3–1.5 mL), pressure (normal range: [-]100–100 daPa), gradient (normal range: <150 daPa), tympanogram type (Jerger’s classification: A, B, C1, C2).

Tympanograms B and C2 were interpreted as OME. Tympanogram C1 was interpreted as a regurgitation of the ET. Normalization was defined as a conversion from B/C1/C2 to A and B/C2 to C1 tympanogram. A transition from B to C2 was interpreted as improvement, and a transition from C1/C2 to B or from A to B/C1/C2 was interpreted as deterioration. The persistence of the same tympanogram type was interpreted as no change. The patients with normalization and improvement were considered responders, while those with no change or deterioration were considered non-responders.

### 2.4. Statistical Analysis

We described patients’ baseline characteristics using a median and interquartile range for continuous and percentages for categorical variables. First, at the participant level, we described a proportion of patients achieving response to AMSA treatment stratified by completeness and laterality. Then, the tympanometry data were analyzed using the affected ear as a unit of analysis (not individual patients—ear-level analysis). Such an approach was adopted in several previous studies [9,11]. We used the McNemar-Bowker test to compare the distribution of tympanogram type before and after AMSA administration (3 x 4 change table A/B/C1/C2). Further, we adopted the Sankey diagram to visualize patterns of tympanogram changes. Continuous changes in volume, compliance, pressure and gradient were assessed using the Wilcoxon signed rank test. Moreover, we compared the percentage of ears achieving a normal range of compliance and pressure before and after the intervention using the McNemar test. Univariable and multivariable-adjusted logistic regression models were adopted to evaluate the association of age, sex, and year season with the prevalence of response to the treatment. To account for the redundancy of patients with bilateral OME, we assigned weights of 0.5 for this group and 1 for unilateral OME.

We used two-sided *p* < 0.05 to denote statistical significance. All analyses were conducted in R version 4.0.5 (R Foundation for Statistical Computing, Vienna, Austria).

## 3. Results

The baseline characteristics of study participants is summarized in Table 1. Among 36 patients included in this study (50 ears affected by OME), 24 (67%) were female, and 12 (33%) were male. The mean age of participants was 51.4 (SD 15.5) years. Twenty-two (61%) and 14 (39%) had, respectively, unilateral and bilateral OME. Affected ears had predominantly B tympanogram 41 (82%), followed by C1 and C2 types (respectively, 6 [12%] and 3 [6%]).

All patients completed the intervention and participated in the follow-up visit. No major adverse events were reported.

**Figure 2:**
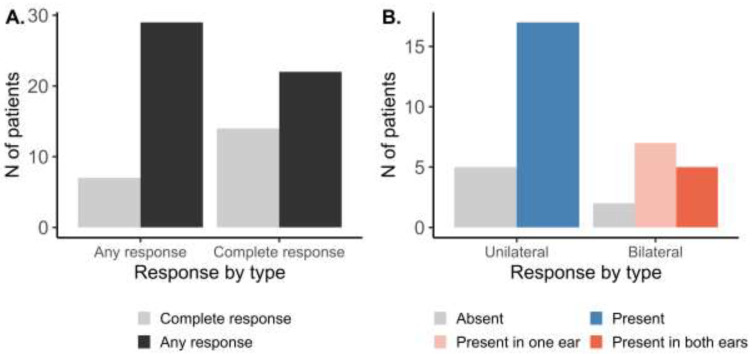
Patient level response to the AMSA treatment stratified by (**A**) completeness or (**B**) laterality of OME.

At the patient level (Figure 2A), following the AMSA procedure, any response (at least in one ear) was observed in 29 out of 36 patients (81%). Complete response (in both ears) was found in 14 patients (39%). Further stratifying the patient-level response by OME laterality (Figure 2B), response to the AMSA procedure was present in 17 out of 22 patients (77%) with unilateral OME. In patients with bilateral OME, response in one of the ears was observed in seven out of 14 patients (50%), while the complete response was observed in 5 of them (14%).

**Figure 3:**
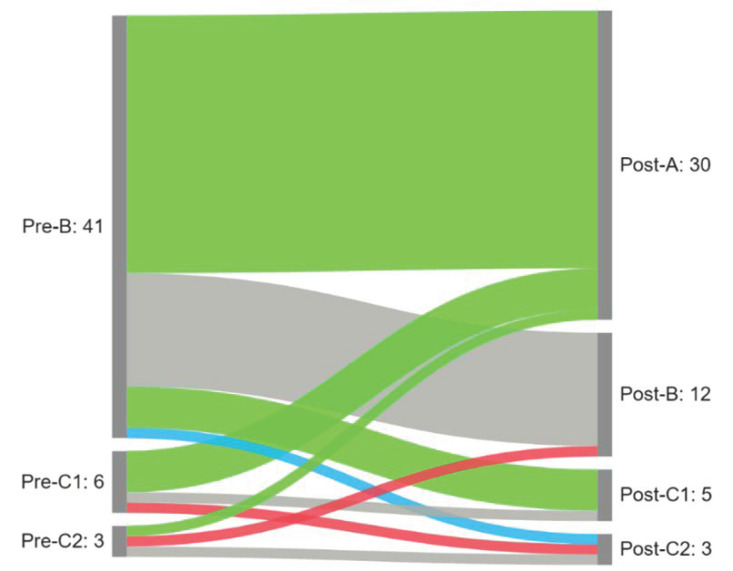
Sankey diagram summarizing patterns of tympanogram change following the intervention (green—normalization; blue—improvement; gray—no changes; red—deterioration).

We observed a significant difference in the distribution of tympanogram types following the intervention (*p* < 0.001). A normalization (conversion from B/C1/C2 to A and B/C2 to C1) was observed in 34 (68%) of the affected ears. An improvement (shift from B to C2) was observed in one ear (2%) and a deterioration in two ears (4%). No change was observed in 13 ears (26%). Results are presented in Figure 3.

Analyses for continuous parameters showed an increase in the volume of the middle ear (∆_median_ 0.19 mL, *p* = 0.002) and a reduction of the pressure in the middle ear (∆_median_ 142 daPa, *p* < 0.001) after the treatment. Results are presented in Table 2. No statistically significant changes were observed for compliance and gradient (*p* = 0.4 and *p* = 0.06, respectively). The proportion of patients achieving compliance and pressure goals was also significantly higher following the treatment (both *p* < 0.001).

None of the proposed effect modifiers (age, sex, and season) had a statistically significant effect on treatment efficacy (all *p* > 0.05). Odds ratios with 95% confidence intervals for the univariable and multivariable models are shown in Table 3.

## 4. Discussion

To the best of our knowledge, this study is the first trial of inhaled mucolytics and steroids in adults with OME, in whom tympanometry was assessed at baseline and after treatment. We found high rates of improvement in tympanometry after three days of treatment. Following AMSA administration, we observed significant improvement in volume, pressure, and compliance measured in tympanometry. No significant differences in response to treatment were observed for patients’ age, sex, or season of inclusion.

A short course of AMSA with a nebulized mucolytic drug and steroid in OME could be regarded as an effective treatment alternative. Moreover, it avoids more disruptive and costly interventions such as myringotomy and grommet insertion. Previous research showed some evidence in favor of AMSA use for OME treatment in children under the age of 18 years. The characteristics and results of previously published studies using AMSA are summarized in Table 4.

These studies vary in the duration of treatment, medication, and outcome assessment methods. In contrast to the other studies that included children, as noted previously, our focus is on the adult population. While the length of intervention in previous studies was at least 5 days, our study demonstrated that a three-day course may also result in a clinical benefit. The reduced duration of treatment was possible due to the adults’ facilitated cooperation and more comprehensive implementation of instructions as compared to the children. Each of the previous studies used a different medication protocol—mucolytics alone [9] or combined with a steroid [8] or an antibiotic with a steroid [10]. In previous studies, aerosols were obtained by mixing two substances, whereas we administered two separate aerosols with other solutions—first a mucolytic, followed by a second steroid-based one. The therapeutic effect was measured differently, and it cannot be compared between the trials. A retrospective study by Saga et al. focused only on pure tone audiometry thresholds. In two of the three mentioned papers, one of the assessment methods was impedance tympanometry as well, but only the tympanogram type was registered. Moreover, in the study by Szkiełkowska et al. [9], the tympanogram after the treatment was not analyzed and the inclusion criterion was type B at least in one ear [9]. Another study [8], by Wilhelmsen, noted an improvement in tympanometry after one week (before: A 0%, B 27%, C 73%; after: A 7%, B 20%, C 73%) We noted the normalization in 68% and improvement in 2% of all affected ears. This study also measured other tympanometry parameters—gradient, pressure, volume, and compliance. In our opinion, this speaks to the robustness of our results. However, we accepted tympanogram type B, C1, and C2, as a diagnostic feature of OME, whereas the actual presence of fluid in the middle ear is not so frequent in type C1 and C2. The C type tympanogram generally indicates ET dysfunction. According to Rosenfeld et al., middle ear effusion is present in 85–100% of type B, 17% of type C1, and 55% of C2 tympanogram of patients at the myringotomy [4,12]. In our study, ears with a pre-intervention type C1/2 tympanogram account for 18% of the study group, which is a relatively small percentage, that does not affect the overall result.

The drugs applied in AMSA are generally steroids combined with a mucolytic drug, saline, or an antibiotic. Locally applied steroids via pulsatile airflow have been shown to facilitate ventilation efficiency and ensure appropriate medication distribution to hard-to-reach areas [13]. Mucolytics, as in our study ambroxol, dissolve and diminish the viscosity of thick mucus in the middle ear. They reduce mucus production, promote its excretion, and decrease inflammation [14]. The steroid and the ambroxol were both dissolved in saline in our study. We, therefore, expect that by doing so, we obtained a more effective flushing of the middle ear and the ET, compared to inhaling undiluted drug solutions. Due to the analogic recommendation on rhinosinusitis, where flushing with saline solution is the basic protocol for any inflammation status, in our opinion, this protocol may be more effective [15]. The treatment is similar in both rhinosinusitis and OME, and in the former case, AMSA simplifies the administration of the solution, as the middle ear is a more difficult-to-reach area. A recently published systemic review by Brescia et al. showed that chronic rhinosinusitis (CRS) is significantly associated with chronic otitis media in 20,867 patients with a diagnosis of CRS, 991 (4.75%), who were also diagnosed with this condition. Therefore, the inflammatory process involves the epithelium in the middle ear and upper airway [16]. Conservative and surgical treatment of CRS may result in the OME resolution.

Studies adopting Politzer insufflations or nasal lavage can be a source of concepts for new AMSA drug protocols. Mirandola et al. reported a clinical improvement of OME in children after Politzer insufflations of sulphurous water, which they compared to the untreated control group [17]. Another randomized control trial demonstrated the clinical improvement of OME in children treated with hyaluronic acid with a hypertonic solution via nasal lavage. Moreover, a significant reduction in the consumption of steroids and antibiotics was observed during the follow-up in this study [18]. New treatment protocols for AMSA can be developed on the basis of these results.

No single perfect method for evaluating the state of the middle ear currently exists. Most studies use pure tone audiometry [17,19], tympanometry [20,21], otoscopy [18], or a combination of the above [11,22]. All the studies mentioned concentrate on the management of OME and evaluate the state of the middle ear before and after the intervention. We have chosen bare tympanometry for its advantages of being short in time, simplicity, and widespread accessibility. The outcomes are both objective and comparable. Pure tone audiometry provides a subjective result, which may vary depending on the patient’s condition, and the degree of concentration. Hence, it does not deliver data on the pressure and condition of the ET. On the contrary, otoscopy is subjective, unaccountable, and highly dependent on the examiner.

The condition of the ET has been linked to OME pathogenesis [23]. A new hypothetical mechanism of the transient sequential ET ventilation contributes evidence proving masticatory muscles’ fundamental role in ET functioning, especially the pterygoid muscle [24,25]. ET rehabilitation includes, among others, muscle-strengthening exercises and insufflation and may be considered a useful therapy in the management of OME [26,27]. One of the methods applied in daily practice is children chewing gum during aerosol therapy to improve drug delivery. Mastication increases the swallowing rate and the rate of paratubular muscle activation and ET opening, which could also be implemented in future studies in adults to enhance the beneficial outcome of AMSA [26].

Our study is not free of limitations. First, this study did not involve a control group. Secondly, the study sample was relatively small, but its pilot character explains this. Patients did not receive a disease-specific quality-of-life questionnaire, nor undergo imaging to exclude nasopharyngeal or anterior/middle cranial fossa pathology, possibly impairing ET function. No pure tone audiometry nor another hearing test before and after the intervention were performed. Moreover, we did not consider a long-term follow-up visit after the end of the intervention. Clearly, larger, controlled clinical studies need to be conducted to confirm our results.

An undoubtful strength of this study is that it is the first report of AMSA use in OME management in adults. Observed effect sizes can be further utilized in the calculation of a minimum sample size in future studies.

## 5. Conclusions

A 3-day course of mucolytics and steroid administration with AMSA was reported to be relatively effective for symptom withdrawal in adults with OME. Partial and complete OME remission was noted in, respectively, 81% and 39% of patients. Moreover, an increase in volume and pressure in the middle ear were observed following the treatment. Treatment efficacy did not depend on patients’ age, sex, or the season of inclusion in the trial. The results of this pilot study are encouraging; however, the use of AMSA management of OME in adults needs to be verified in future studies.

## Figures and Tables

**Table 1:** Baseline characteristics of patients by treatment group.

Characteristic	*n* (%)
N of patients	36 (100%)
Age at recruitment (years)	51.4 (15.5) ^1^
Gender: Male Female	12 (33%) 24 (67%)
Season of inclusion: Spring (March–May) Summer (June–August) Autumn (September–November) Winter (December–February)	11 (31%) 3 (8%) 10 (28%) 12 (33%)
Laterality	
Unilateral	22 (61%)
Bilateral	14 (39%)
N of affected ears	50 (100%)
Pre-intervention tympanogram	
A	0 (0%)
B	41(82%)
C1	6 (12%)
C2	3 (6%)

^1^ Mean (Standard Deviation).

**Table 2:** Tympanometry parameters expressed as continuous and categorical variables before and after the administration of the intervention.

Parameter	Before Intervention	After Intervention	∆median	*p*
Continuous ^1^				
Volume (mL)	0.96 (0.75, 1.15)	1.15 (0.88, 1.46)	0.19	0.002
Compliance (mL)	0.31 (0.22, 0.50)	0.39 (0.30, 0.64)	0.08	0.4
Pressure (daPa)	−172 (−199, −67)	−30 (−118, 16)	142	<0.001
Gradient (daPa)	168 (107, 190)	136 (94, 168)	−32	0.06
Categorical ^2^				
Compliance 0.3–1.5 mL	8 (16%)	27 (54%)	-	<0.001
Pressure −100–100 daPa	14 (28%)	32 (64%)	-	<0.001

^1^ Values for continuous parameters are medians with interquartile ranges. ^2^ Values for categorical parameters are numbers with percentages. P—P for Wilcoxon paired test (continuous parameters) or McNemar’s test (categorical parameters).

**Table 3:** Differences in curve normalization by baseline characteristics.

Parameter	Univariable Model			Multivariable Model		
	OR	95% CI	*p*	OR	95% CI	*p*
Age (years)	0.99	0.96, 1.03	0.7	0.99	0.96, 1.03	0.7
Sex						
Female	ref.			ref.		
Male	1.31	0.48, 3.79	0.6	2.88	0.75, 13.4	0.14
Season						
Fall	Ref.			Ref.		
Summer	0.21	0.02, 1.40	0.12	0.12	0.01, 1.27	0.088
Spring	0.51	0.14, 1.80	0.3	0.55	0.12, 2.37	0.4
Winter	1.04	0.28, 3.85	>0.9	1.21	0.30, 4.95	0.8

The univariable model included age, sex, and season separately, while multivariable model included all three variables in the same model. OR—odds ratio, CI—confidence interval;.

**Table 4:** Summary of previously published interventional studies utilizing ASMA inhalation.

Author Year Country	Age	N of Patients Treated with AMSA	Medication (Length of Treatment)	Tympanogram at Entry	Additional Tests at Entry	Main Results
Szkiełkowska 2005 Poland [9]	3–7	50 (11 unilateral, 39 bilateral OMS)	Ambroxol 7.5 mg + 5 mL saline (5 days; 2 x daily)	Type B at least in one ear	Otoscopy	Clinical improvement in videotoscopy and tympanometry
Saga 2009 Spain [10]	4–15	37	Clindamycin Methylprednisolone Mesnum (6–30 days; mean: 12)	-	Pure tone Audiometry	Clinical Improvement in PTA
Wilhelmsen 2016 Poland [8]	4–17	30	Pulmicort 0.25 mL + N-acetylcysteine 1 mL (5 days 2 x daily)	Type C 73% Type B 27%	Pure tone Audiometry	Clinical improvement in PTA, tympanometry, and stapedius muscle threshold

## Data Availability

Data available on request due to restrictions, e.g., privacy or ethics. The data presented in this study are available on request from the corresponding author. The data are not publicly available due to the protection of personal data.
